# The International Eugenic Conference

**Published:** 1912-12

**Authors:** 


### The International Eugenic Conference.

It is striking to note that at the International Eugenic Conference, held in London, Dr. Pearson, the foremost exponent of eugenics, did not attend inasmuch as he regarded the subjects upon the program for discussion to be still sufficiently moot, to be kept in the laboratory for further investigation.

The disadvantage of premature discussion was shown. All the intrinsic questions relating to race betterment were treated and there was manifest in the varying opinions expressed at the conference a lack of sufficient data necessary to formulate a thorough and well-defined eugenic platform. Mendelism does not enter into all phases of heredity. Its full scope and limitations are not known. Years of investigation will be required before the influence of the Mendelian principle can be demonstrated. The most vital debate of the conference related to the probable value of limiting marriage through State regulations as opposed to the single step of segregation of the unfit.

The total result in positive terms of the conference was the demonstration of the necessity of patience and calm judgment in investigating eugenic questions, before entering into legislation that might tend to retard the general progress of the movement. "Fools rush in where angels fear to tread," and the race must be protected from unscientific theories, crystallized in unfortunate and unwise laws.

The facts relating to heredity must be secured. The influence of disease and excess must be proven beyond cavil. The true eugenist pleads that remedial measures must be established upon a foundation that has no weak points. For upon the foundation depends the strength of the structure to be reared.—*Medical Review of Reviews.*

